# A parallel approximate string matching under Levenshtein distance on graphics processing units using warp-shuffle operations

**DOI:** 10.1371/journal.pone.0186251

**Published:** 2017-10-10

**Authors:** ThienLuan Ho, Seung-Rohk Oh, HyunJin Kim

**Affiliations:** School of Electronics and Electrical Engineering, Dankook University, Yongin-si, Republic of Korea; Tianjin University, CHINA

## Abstract

Approximate string matching with *k*-differences has a number of practical applications, ranging from pattern recognition to computational biology. This paper proposes an efficient memory-access algorithm for parallel approximate string matching with *k*-differences on Graphics Processing Units (GPUs). In the proposed algorithm, all threads in the same GPUs warp share data using warp-shuffle operation instead of accessing the shared memory. Moreover, we implement the proposed algorithm by exploiting the memory structure of GPUs to optimize its performance. Experiment results for real DNA packages revealed that the performance of the proposed algorithm and its implementation archived up to 122.64 and 1.53 times compared to that of sequential algorithm on CPU and previous parallel approximate string matching algorithm on GPUs, respectively.

## Introduction

Approximate string matching (ASM) has been widely applied in many fields, including network intrusion detection systems, voice recognition, web searching, and computational biology [1–3]. Basically, ASM is the problem of finding all positions of a string where a given pattern occurs, allowing a limited number of *errors* in the matches. The closeness of a match is measured by the minimum number of edit operations used to convert a factor of the input string into an exact match of the pattern. The usual edit operations are insertion, deletion, replacement, and transposition [1, 4]. There is a popular method for ASM that allows three edit operations of insertion, deletion, and substitution to transform a factor of the input string into the pattern, which is called ASM with differences. This method is also called as ASM with edit distances or ASM with Levenshtein distance [5].

In order to improving the ASM computation, several techniques have been proposed by adopting field-programmable gate array (FPGA) [6–9], central processing unit (CPU) [10–12], multi-core processors [13], and GPUs [4, 14–18]. The GPU-based ASM provides great parallelism with a large number of threads compared to multi-core processors. Moreover, GPUs have low cost, high flexibility, and updatability, compared to FPGA. In recent years, NVIDIA has introduced CUDA (Compute Unified Device Architecture) with a new parallel programming model and instruction set architecture. CUDA provides a complete tool to leverage the parallel compute engine in NVIDIA’s GPUs. GPUs and their CUDA create new opportunities to study or improve current tools and algorithms.

Traditional sequential algorithms for ASM with *k*-differences adopt the dynamic programming model. These algorithms can compute edit distance matrix *D* between input string and pattern to get the minimum edit operations to convert each factor of input string to pattern. Unfortunately, each element of the matrix *D* depends on the former elements on the same row, or column. Therefore, it is difficult to design a parallel algorithms for ASM with *k*-differences.

Previous researches [16, 17, 19–23] proposed several parallel algorithms for ASM with *k*-differences on CREW-PRAM model and GPUs. The common idea of these researches was that all elements in the same diagonal of the edit distance matrix could be computed in parallel. The paper [20] presented an implementation of ASM algorithm with the hierarchical memory machine (HMM) model on GPUs. The HMM model could capture the essential feature of memory access of NVIDIA GPUs to enhance the performance of ASM algorithm. In [21], a hybrid CPU-GPU model to accelerate ASM algorithm for protein sequences was proposed. The main goal was to speed up computational time of ASM algorithms using a hybrid model that combines the power of multicore CPUs and that of contemporary GPUs. In this case, CPUs read the task and control the number of queries that were sent to GPUs. CPUs were processed in parallel to enhance the reading task from multiple files. This hybrid model should be adopted in the architecture of NVIDIA GPUs with computing capability 3.0 or higher, which allows CPUs to send multiple jobs to GPUs. The research [22] presented a hybrid approach that combines ASM with *k*-differences algorithm and Needleman–Wunsch (NW) algorithm in order to find similarities of two long protein sequences. In this case, ASM with *k*-differences algorithm was used to calculate the edit distances between pairs of parts of protein sequences. When comparing two sequences, the NW algorithm was used to combine with some scored edit distances to compute the final edit distance which can finally indicate the similarity level. The hybrid approach of [22] was implemented in a parallelism model by coupling CPU multi-threaded operations into GPU algorithm to maximize performance. In addition, database storage matrix was transposed based on the ELL (Ellpack-Itpack) storage format for coalesced memory access of GPU threads. Thus, the proposed parallel approach could achieve high compute and storage efficiency. In [23], a heterogeneous CPU-GPU computing system for measuring the similarity between RNA/DNA sequences was presented. In CPU-GPU implementation, CPU handled work assignment and data distribution while GPU was responsible for the whole parallel computation. To fully utilize the computing devices, the proposed system took a co-run computation model so that workloads were assigned and computed on both CPU and GPU devices simultaneously. In addition, a pre-computation mechanism was developed to distribute workloads to CPU and GPU based on their computing capacity. The computing system of [23] could maximize the utilization of computing resources to enhance performance. The works of paper [24, 25] presented several approaches for checking the similarity between the large-scale data of RNA/DNA sequences. The proposed approaches were developed based on MapReduce model with Apache Hadoop platform [26]. These approaches have archived significant performance and scalability. However, there was a problem of combining GPUs and Hadoop platform. In this case, GPUs should be driven in the local disk. In cloud computing system of Hadhoop, the graphic memory could not be found since the NVIDIA graphic card was not driven. The combination of GPU and Hadhoop has been proposed to be a promising field by solving technique problem encountered by NVIDIA company and Apache [24]. In addition, adopting MapReduce model to CPU-GPU systems without using Apache Hadoop platform has been demonstrated previously [24, 27]. In this case, the long RNA/DNA sequences were split and sent to different client nodes. In each client node, a couple of CPU and GPU were combined to process for each part of the RNA/DNA sequence in parallel. Based on this idea, the paper [27] proposed a hybrid scheduling technique for GPU-based computer clusters. A scheduling technique was developed by extending Hadoop procedure to minimize the execution time of a submitted job on CPU cores and GPU devices. Thus, the total execution time could be reduced. Two implementations of parallel ASM for DNA sequencing on GPUs relying on warp-shuffle operations were proposed in [16, 17]. The main parallel scheme was the same as that in previous studies [19, 20] that processed the edit distance matrix in parallel for all elements in the same diagonal flow. Moreover, the papers [16, 17] adopted wrap-shuffle operations to reduce the communication overhead between threads. By parallel computing for all elements in the same diagonal flow, the maximum number of threads processed in the same time was limited by the length of the input pattern. Therefore, the parallelism scheme in [16, 17, 19] lacked the parallel expandability, especially when adopting GPUs.

The paper in [4] proposed an algorithm with a parallel scheme difference from the works in [16, 17, 19], where all elements in the same row of edit distance matrix could be calculated in parallel by eliminating data dependency. This parallel scheme achieved the maximum number of threads processed in the same time up to the length of input string. In addition, the length of input string was much longer than that of pattern. Compared to the diagonal parallel scheme, the row parallel scheme in [4] could thoroughly use processors when the number of available processors was great, especially when using GPUs.

This paper proposes an efficient memory-access algorithm and its implementation for parallel ASM with *k*-differences on GPUs. Unlike the achievement in [4], to enhance the throughput, all threads in the same warp can communicate with each other using the warp-shuffle operations without accessing the memory. In this case, by using warp-shuffle operations, our proposed algorithm can reduce the amount of read/write operations when computing all elements in the edit distance matrix. In order to overcome the limitation of warp-shuffle operations when they cannot be used for communication between threads in neighboring warps, the proposed implementation adopts write/read operations in shared memory or global memory instead of using warp-shuffle operations. In the particular case of adopting GPUs, the proposed algorithm can be implemented by exploiting memory structure of GPUs to enhance its performance. The performance of the proposed approach is measured using random DNA pattern from [28] and several input strings from raw DNA sequences [29]. Results of experiments show that the performance of the proposed algorithm and its implementation is enhanced up to 122.64 and 1.53 times, compared to that of sequential algorithm implemented on CPU and previous parallel ASM algorithm on GPUs, respectively.

The rest of this paper is organized as follows. *Previous Works* section explains the problem of ASM with *k*-differences. Traditional sequential algorithm and previous parallel algorithm for solving ASM with *k*-differences are then shown. The details of the proposed algorithm and its implementation are described next. Finally, experiments and performance comparisons are discussed.

## Previous works

### Definition 1: Edit distance

For two strings *X*[0…*n* − 1] and *Y*[0…*m* − 1], the edit distance between *X* and *Y*, denoted as *ED*(*X*, *Y*), is the minimum number of edit operations used to convert string *X* to *Y*. There are three edit operations defined as follows:

- Insert a character to *X*.
- Delete a character from *X*.
- Replace a character of *X*.

### Definition 2: ASM with *k*-differences

Given a string *T*[0…*n* − 1], a pattern *P*[0…*m* − 1], and a threshold *k*, where *n* > *m* > *k*, ASM with *k*-mismatches searches for all factors *u* of length *m* in *T* that is formulated by *ED*(*u*, *P*) ≤ *k*.

### Traditional sequential algorithm for computing edit distance

Traditional sequential algorithm for solving ASM with *k*-differences is developed based on dynamic programming model. In this case, the edit distance matrix *D* of input string *T* and pattern *P* is calculated, where each element contains the minimum edit operations between a factor of *T* and a factor of *P*.

Given *i*, *j* (0 ≤ *i* ≤ *m*, 0 ≤ *j* ≤ *n*), the element *D*[*i*, *j*] of the edit distance matrix *D* can be calculated by (1).

$$D\left[i,j\right]=\{\begin{array}{cc} 0, & \text{if}\;i=0\\ i, & \text{if}\;j=0\\ D\left[i-1,j-1\right], & \text{if}\;T\left[j-1]=P[i-1\right]\\ 1+min\left\{D\left[i-1,j],D[i,j-1],D[i-1,j-1\right]\right\}, & \text{otherwise}. \end{array}$$ (1)

**Fig 1:**
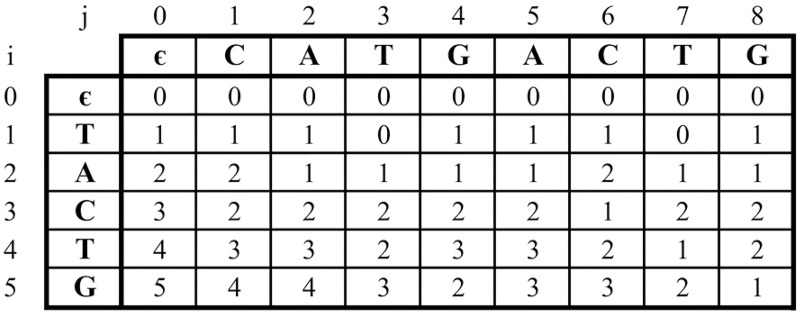
Edit distance matrix *D* for input string *T* = *CATGACTG* and pattern *P* = *TACTG*.

Fig 1 shows an example of edit distance matrix, where input string *T* = *CATGACTG*, pattern *P* = *TACTG*, and threshold *k* = 2. In this case, each element *D*[*i*, *j*] contains the minimum edit operations, which are used to convert factor *T*[*l*…*j* − 1] to *P*[0…*i* − 1] (0 ≤ *l* ≤ *j* − 1).

To compute edit distance matrix *D* by using (1), the value of *D*[*i*, *j*] depends on its previous elements *D*[*i*, *j* − 1], *D*[*i* − 1, *j*], and *D*[*i* − 1, *j* − 1]. Data dependency appears in the traditional sequential algorithm. Therefore, it is difficult to develop a parallel algorithms for ASM with *k*-differences.

### Previous parallel algorithm for ASM with *k*-differences

To enhance the performance of ASM with *k*-differences, several parallel algorithms have been developed in [16, 17, 19]. The main idea is based on the parallelism when all elements in the same diagonal of the edit distance matrix *D* are computed in parallel. In this case, the maximal number of threads processed at the same time is *m* + 1, where *m* is length of target pattern. Therefore, these approaches cannot fully use threads when the number of available threads is great.

To expand the number of threads that can process the edit distance matrix at the same time, the paper [4] presented a technique for eliminating data dependency. In this case, a matrix *X* of dimension |∑| * (*n* + 1) is constructed, where |∑| and *n* are size of character set ∑ and length of input string *T*, respectively. Array *Q*[0, …, |∑| − 1] denotes characters in ∑. *X*[*i*, *j*] can be calculated as (2).

$$X\left[i,j\right]=\{\begin{array}{cc} 0, & \text{if}\;i=0\\ j, & \text{if}\;T\left[j-1]=Q[i\right]\\ X\left[i,j-1\right], & \text{otherwise}. \end{array}$$ (2)

**Algorithm 1** Parallel algorithms for ASM with *k*-differences.

1: Input: *T*, *P*, *n*, *m*

2: Output: Edit distance matrix *D*

3: **begin**

4: **for all** *i* ∈ ∑ **parallel do**

5:  **for** *j* ← 0 **to** *n* **do**

6:   Compute matrix *X* using (2).

7:  **end for**

8: **end for**

9: **for all** *i* ← 0 **to** *m* **do**

10:  **for** *j* ← 0 **to** *n* **parallel do**

11:   Compute edit distance matrix *D* using (3).

12:  **end for**

13: **end for**

14: **end**

**Fig 2:**
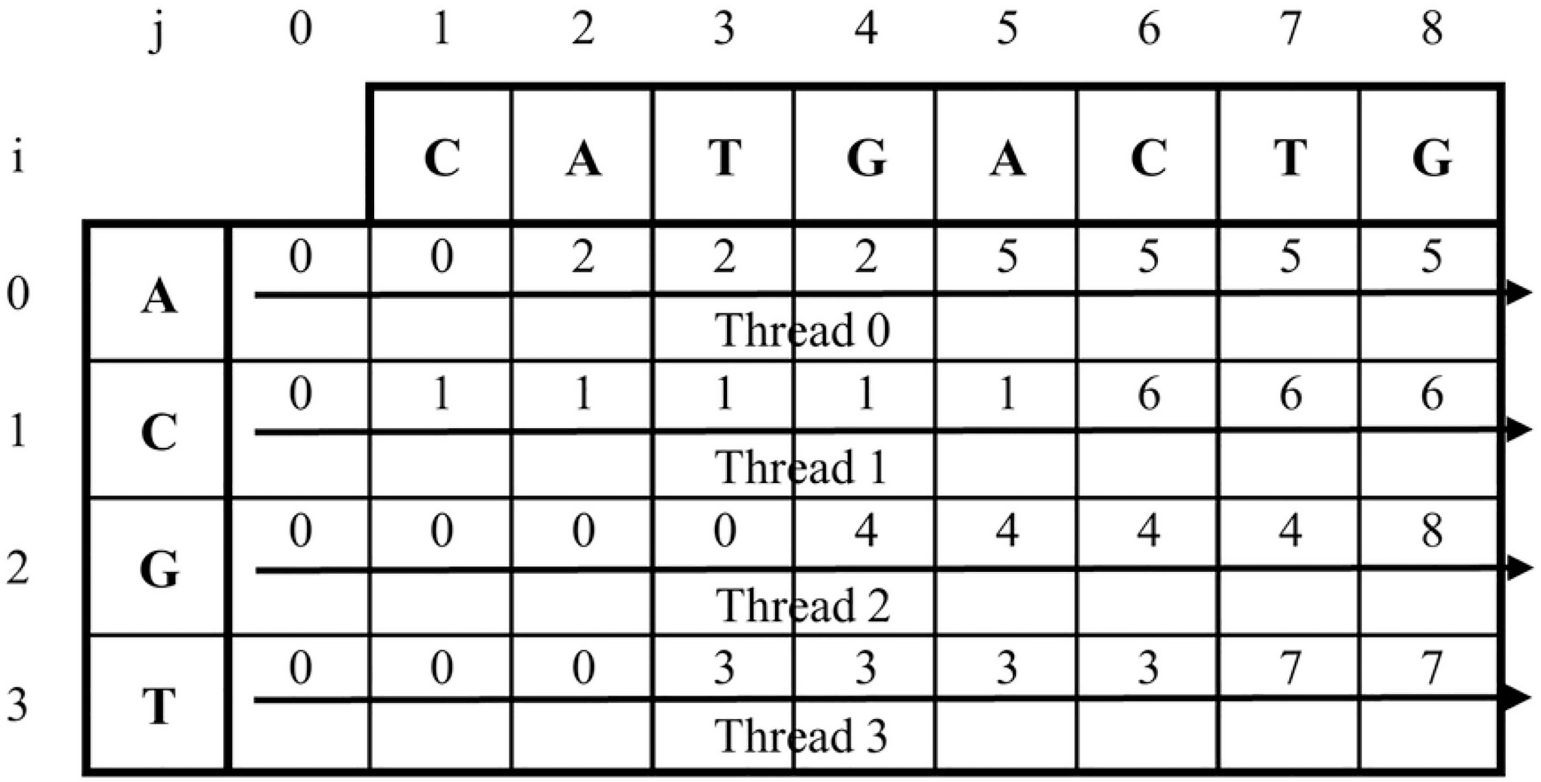
Matrix *X* of input string *T* = *CATGACTG*.

Fig 2 shows an example of matrix *X* of the input string *T* = *CATGACTG*. In this case, data of all rows are independent from each other. Therefore, all rows of matrix *X* can be computed in parallel.

According to matrix *X*, (1) can be written as follows.

$$D\left[i,j\right]=\{\begin{array}{cc} 0, & \text{if}\;i=0\\ i, & \text{if}\;j=0\\ D\left[i-1,j-1\right], & \text{if}\;T\left[j-1]=P[i-1\right]\\ 1+min\left\{D[i-1,\;j],\;D[i-1,\;j-1],\;i+j-1\right\}, & \text{if}\;X[l,\;j]=0\\ 1+min\{D[i-1,\;j],\;D[i-1,\;j-1], & \\ D[i-1,\;X[l,\;j]-1]+(j-1-X[l,\;j])\}, & \text{otherwise}. \end{array}$$ (3)

In (3), *l* is the location of *P*[*i* − 1] (1 ≤ *i* ≤ *m*) in array *Q*. Considering (3), data in the *i*-th row of *D* only depends on data from the (*i* − 1)-th row. Therefore, the parallel of all elements in each row of edit distance matrix *D* can be computed in parallel. However, the computation of all elements in the same row should be completed before the processing of next row. The pseudo code of previous parallel ASM with *k*-differences is described as Algorithm 1.

**Fig 3:**
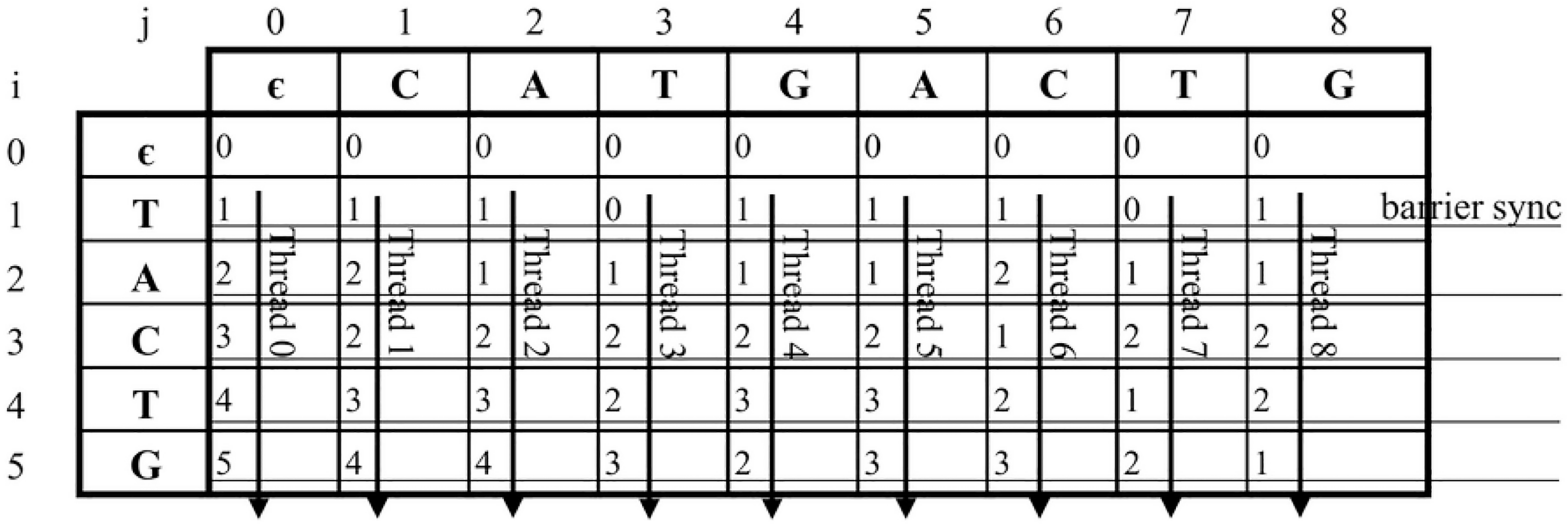
An example of Algorithm 1 for input string *T* = *CATGACTG* and pattern *P* = *TACTG*.

Fig 3 shows an example of adopting algorithm 1 for ASM with *k*-differences with input string *T* = *CATGACTG*, target pattern *P* = *TACTG*, and threshold *k* = 2. Threads are assigned to process all elements in the same row of the edit distance matrix *D*. The *barrier sync* is called to guarantee that all threads can complete processing all elements of a row before going to the next row. Finally, all elements in the *m*-th row are compared with *k* to store into array *Result*[]. For all *j*, 0 ≤ *j* ≤ *n* − 1, if *D*[*m*, *j* + 1] ≤ *k*, *Result*[*j*] = *j*; otherwise, *Result*[] = −1.

## Proposed parallel algorithm and its implementation for ASM with *k*-differences on GPUs

This section proposes an efficient memory-access parallel algorithm and its implementation for ASM with *k*-differences on GPUs. The key idea of the proposed algorithm is based on adopting warp-shuffle operations to eliminate the assesses of global or shared memories. Moreover, the proposed algorithm is implemented by exploiting the memory structure of GPUs to optimize its performance. Details of warp-shuffle operation and the implementation procedure are explained in this section.

### Warp-shuffle operations on GPUs

NVIDIA has proposed a parallel computing platform and programming model, called CUDA. It is adopted to leverage the parallel compute engine in GPUs to solve many complex computational problems with great parallelism [18, 30–32]. A CUDA contains a thousand general purpose computing processors, named *threads*. The architecture of CUDA threads is organized by *blocks* and *grids*. A block is a group of threads while a grid is a group of blocks. Each grid is assigned to process a core program, named *kernel*, which is provided by host/CPU. In CUDA, all blocks in a grid can be executed in parallel. However, there is no communication between blocks [18, 30]. In a block, threads are organized into several groups of threads, called *warps*, to execute the same instruction. Typically, all threads in the same block can communicate with each other by using *shared memory*. In addition, recent NVIDIA GPU architecture with computing capability 3.0 or higher provides a way to share data between threads in the same warp directly. In this case, a thread can read registers of other threads in the same warp by using a new instruction called *warp-shuffle* [16, 17, 33]. There are four warp-shuffle operations, named __*shfl*(), __*shfl*_*down*(), __*shfl*_*up*(), and __*shfl*_*xor*(), to support threads within a warp to collectively exchange or broadcast data.

Threads within a warp are referred to as *lanes*. They are indexed from 0 to *w* − 1, where *w* is the number of threads in a warp. Considering the warp-shuffle function __*shfl*_*up*(*par*, *delta*), it calculates a destination lane ID by subtracting *delta* from the caller’s lane ID. The calling thread can then take a variable *par* from the local register of another thread according to destination lane ID. As an example, when a thread calls a warp-shuffle function __*shfl*_*up*(*par*, 1). Because *delta* is 1, the __*shfl*_*up*(*par*, 1) operation allows the calling thread to get the value of variable *par* in local register of the thread whose lane ID is immediately lower than that of the calling thread.

**Fig 4:**
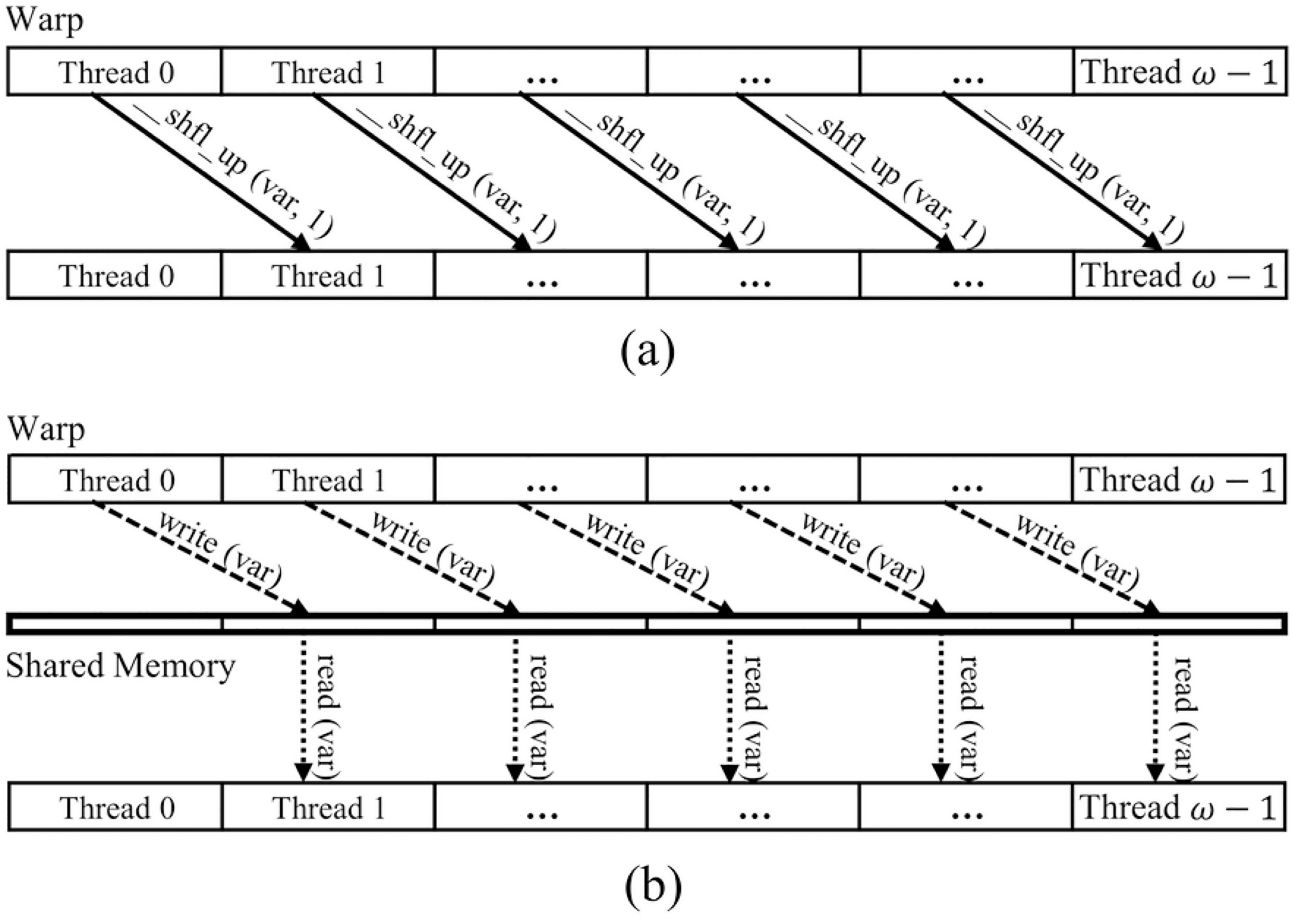
Data transference of threads in a warp: (a) Transferring data via __*shf_up()* operation; (b) Transferring data via alternative write/read operations on shared memory.

Fig 4 shows an example of data transfer between threads in a warp. Fig 4(a) shows data transfer of threads via __*shfl*_*up*(*var*, 1) operation. In this case, thread *i* uses __*shfl*_*up*(*var*, 1) operation to directly get the value of *var* in local register of thread *i* − 1, where 0 < *i* < *w*. On the other hand, Fig 4(b) shows data transfer of threads via alternative write/read operations on shared memory. Each thread writes the value of its variable *var* into the specific location in the shared memory. The synchronize instruction should be called to wait until all threads completely finish the write process. Thread then needs to access the shared memory to get the value of *var* of the thread that has the immediate lower ID.

By using warp-shuffle operations, several advantages can be achieved. Firstly, the shuffle instruction frees up shared memory to be used for other data. Secondly, the shuffle instruction is faster than shared memory since it only requires one instruction versus three instructions for shared memory (write, synchronize, and read). Finally, the shuffle instruction can be used instead of warp-synchronous optimization.

### Proposed parallel algorithm and its implementation for ASM with *k*-differences on GPUs using warp-shuffle operations

In this section, a parallel algorithm and its implementation for ASM with *k*-differences are proposed. The proposed algorithm adopts warp-shuffle operation in order to enhance the performance, compared to that in [4]. By using warp-shuffle operations, all threads in the same warp can transfer data without using write/read/sync operations on shared memory. Therefore, the communication overhead between threads in the same warp can be reduced. Moreover, the proposed algorithm exploits memory structure of GPUs to optimize performance of the previous approach.

In the proposed algorithm, all threads are assigned to process elements in the same row of the edit distance matrix *D* in parallel based on (2) and (3), where thread processing based on warp-shuffle operations is implemented.

Considering (3), the element *D*[*i*, *j*] depends on values of *D*[*i* − 1, *j* − 1], *D*[*i* − 1, *j*], and *D*[*i* − 1, *X*[*l*, *j*] − 1], which are already calculated in the previous row. Therefore, before a thread calculates *D*[*i*, *j*], values of *D*[*i* − 1, *j* − 1], *D*[*i* − 1, *j*], and *D*[*i* − 1, *X*[*l*, *j*] − 1] should be transferred to the thread. Assuming that thread *j* stores its correlative *D*[*i*, *j*] value in its local register *Dvar*, where 0 < *i* ≤ *n*. Let us consider how values *D*[*i* − 1, *j* − 1], *D*[*i* − 1, *j*], and *D*[*i* − 1, *X*[*l*, *j*] − 1] are transferred and stored in register *Avar*, *Bvar*, and *Cvar* of the calling thread, respectively.

- The value of *D*[*i* − 1, *j* − 1] is stored in the register *Dvar* of the thread that has the immediate lower ID. Therefore, __*shfl*_*up*() operation can be used. Therefore, *Avar* = __*shfl*_*up*(*Dvar*, 1)
- The value of *D*[*i* − 1, *j*] is stored in the register *Dvar* of the current thread. Therefore, *Bvar* = *Dvar*.
- The value of *D*[*i* − 1, *X*[*l*, *j*] − 1] is stored in the previous row of the matrix *D*. Therefore, *Cvar* = *D*[*i* − 1, *X*[*l*, *j*] − 1].

Based on values of *Avar*, *Bvar*, and *Cvar*, a thread can calculate the value of *Dvar* by using (4).

$$Dvar=\{\begin{array}{cc} 0, & \text{if}\;i=0\\ i, & \text{if}\;j=0\\ Avar, & \text{if}\;T\left[j-1]=P[i-1\right]\\ 1+min\left\{Avar,Bvar,i+j-1\right\}, & \text{if}\;X\left[l,j\right]=0\\ 1+min\left\{Avar,Bvar,Cvar+\left(j-1-X\left[l,j\right]\right)\right\}, & \text{otherwise}. \end{array}$$ (4)

**Fig 5:**
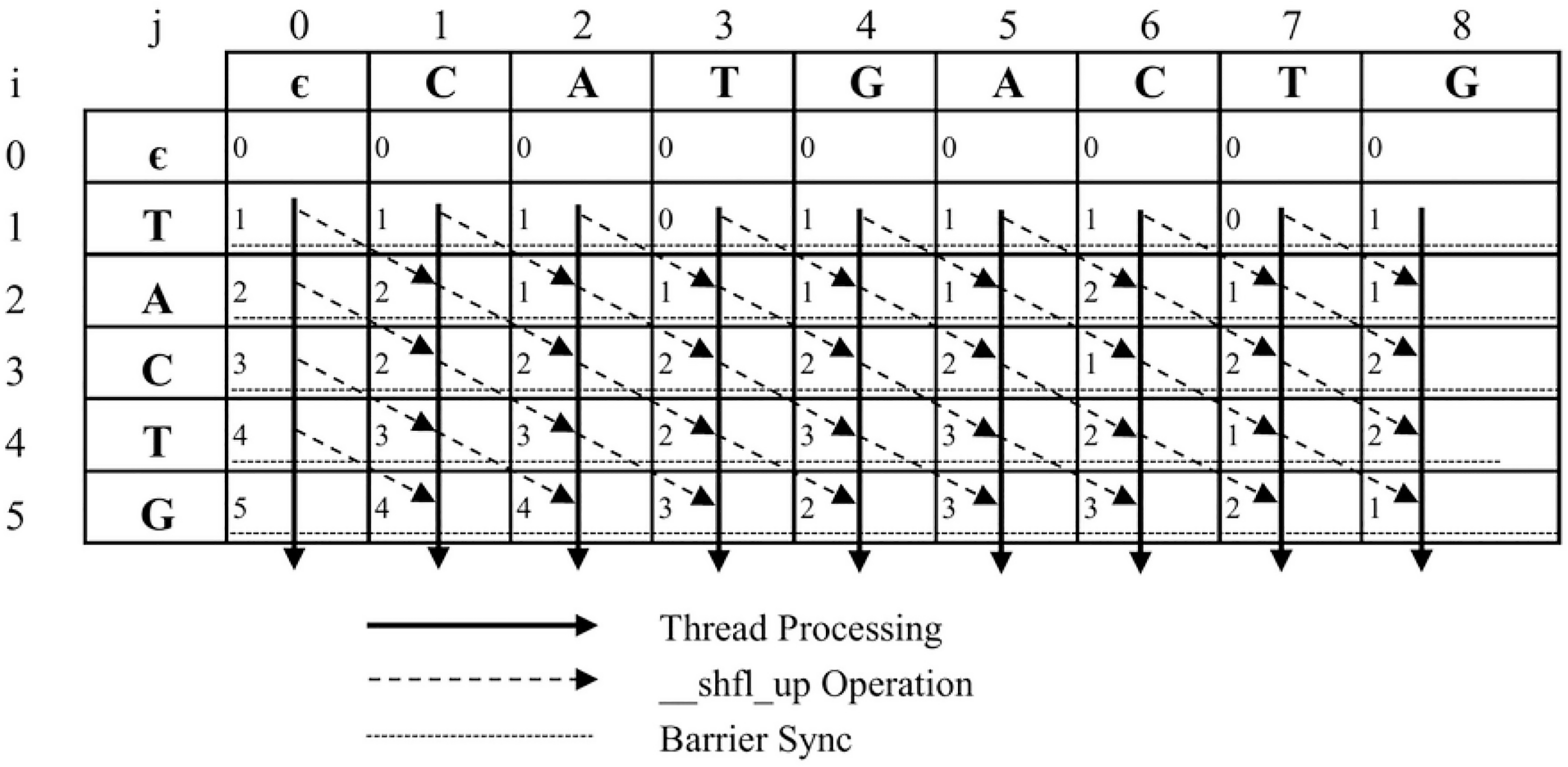
An example of Algorithm 2 for input string *T* = *CATGACTG* and pattern *P* = *TACTG*.

The pseudo code of the proposed algorithm is shown in Algorithm 2. Fig 5 shows an example of adopting algorithm 2 for solving ASM with *k*-differences, where input string *T* = *CATGACTG*, pattern *P* = *TACTG*, and threshold *k* = 2. Threads are assigned to process elements in the same row of the edit distance matrix *D*. Before thread *i* processes its element, a warp-shuffle operation is performed to get the *Dvar* value of thread that has the lane IDs *i* − 1. The *barrier sync* instruction is called to guarantee that all threads can completely process all elements of one row before going to the next row.

**Algorithm 2** Proposed parallel algorithm for ASM with *k*-differences.

1: Input: *T*, *P*, *n*, *m*, *w*

2: Output: Edit distance matrix *D*

3: **begin**

4: **for all** *i* ∈ ∑ **parallel do**

5:  **for** *j* ← 0 **to** *n* **do**

6:   Compute matrix *X* using (2).

7:  **end for**

8: **end for**

9: **for all** *i* ← 0 **to** *m* **do**

10:  **for** *j* ← 0 **to** *n* **parallel do**

11:   **if** *i*%*w* = = 0 **then**

12:    *Avar* = *D*[*i* − 1, *j* − 1];

13:   **else**

14:    *Avar* = __*shfl*_*up*(*Dvar*, 1);

15:   **end if**

16:   *Bvar* = *Dvar*;

17:   *Cvar* = *D*[*i* − 1, *X*[*l*, *j*] − 1];

18:   Compute edit distance matrix *D* using (4).

19:   *D*[*i*, *j*] = *Dvar*;

20:  **end for**

21: **end for**

22: **end**

The limitation of warp-shuffle operations is that data transfer cannot be processed between threads in two neighboring warps. Therefore, the leftmost or rightmost threads in two neighboring warps should communicate with each other using write/read operations in shared memory or global memory.

In addition, the performance of the proposed algorithm is implemented on GPUs by exploiting CUDA memory structure. In CUDA memory architecture, global memory has high-capacity. Shared memory can provide fast memory access for threads. However, the capacity is smaller than that of global memory [30]. With the limitation of shared memory size, one main contribution of our implementation is to find out which access part of global memory to be replaced by that of shared memory. The obtained part of the global memory is transferred to the shared memory of block. By accessing the shared memory instead of the global memory, the execution time of threads can be reduced.

In the implemented version, threads are arranged into blocks to adopts the feature of shared memory. Because threads start from continuous positions of input string, they only need a part of input string for their processing. This part of input string can be called *substring*. The length of substring is equal to the number of threads in one block. All threads in the same block access substring and target pattern frequently. Before the execution of threads, all threads in the same block copy block substring and target pattern into the shared memory. The copy should be done before the processing of threads. Thus, a *__syncthreads()* is used. Threads can then execute ASM in parallel. However, the information of substring and target pattern can be accessed in shared memory instead of global memory.

## Experimental results

In this section, performance of the proposed algorithm and its implementation was evaluated on both NVIDIA GPU GeForce GTX 660 [34] and Intel Xeon CPU E31270 [35]. For realistic experiments, input packets were extracted from [29]. Target patterns were captured from [28]. The performance was presented by execution time, or throughput, which was calculated as (5).

$$\begin{array}{c} Throughput=\frac{Number\;of\;bytes\;in\;input\;string}{Total\;processing\;time} \end{array}$$ (5)

For throughput or execution time comparisons, each approach was denoted as follows.

- ASM_CPU: The sequential ASM algorithm ran on CPU with CPU memory.
- PASM_GPU: The previous parallel ASM algorithm ran on GPU with global memory [4].
- WsPASM_GPU: The proposed parallel ASM algorithm ran on GPU with global memory.
- WsPASM_GPUshared: The proposed parallel ASM algorithm ran on GPU with global memory and shared memory.

Table 1 shows the comparison of proposed and previous approaches with various sizes of input string in terms of throughput. In discussion, there are three points that need to be considered. Firstly, by adopting parallel execution on GPUs, the performance was greatly enhanced, compared to the processing on CPU. In the case of input string of 22.87Mbytes, throughput of ASM_CPU, PASM_GPU, WsPASM_GPU, and WsPASM_GPUshared were estimated to be 0.026, 1.57, 1.84, and 1.99 Gbps, respectively. The performance of PASM_GPU, WsPASM_GPU, and WsPASM_GPUshared archived 60.38, 70.77, and 76.54 times faster than that of ASM_CPU. Secondly, WsPASM_GPUshared exploited the memory model of GPUs to optimize the performance of WsPASM_GPU. With input string of 22.87Mbytes, WsPASM_GPUshared enhanced the throughput by 8.15%, compared to WsPASM_GPU. Thus, WsPASM_GPUshared provided a good example by combining shared memory and global memory of GPUs to optimize the performance. Thirdly, throughput results showed the great performance enhancement of the proposed algorithms, compared to that of previous algorithms. In the case of 7.74Mbytes of input string, throughput values of WsPASM_GPUshared, PASM_GPU, and ASM_CPU were estimated by 1.99, 1.52, and 0.025 Gbps, respectively. Therefore, WsPASM_GPUshared outperformed PASM_GPU and ASM_CPU by 1.31 and 79.6 times, respectively. On average, WsPASM_GPUshared outperformed PASM_GPU and ASM_CPU by 1.25 and 76.26 times, respectively.

**Table 1:** Throughput comparison for several input packets.

info.*				throughput(Gb/s)			
input(Mbytes)	Pat. Length^1^	*k*	Matches^2^	ASM_CPU	PASM_GPU	WsPASM_GPU	WsPASM_GPUshared
3.14			50.57K	0.025	1.59	1.83	1.94
4.87			76.94K	0.026	1.58	1.84	1.95
7.74	16	6	119.90K	0.025	1.52	1.87	1.99
12.88			198.74K	0.026	1.61	1.87	1.98
17.56			269.51K	0.026	1.59	1.84	1.97
22.87			585.16K	0.026	1.57	1.84	1.99

* The table shows throughput results from various sizes of input packets, a target pattern of length of 16, and threshold *k* = 6.

^1^ Length of patterns.

^2^ Number of matched patterns in the input string.

**Fig 6:**
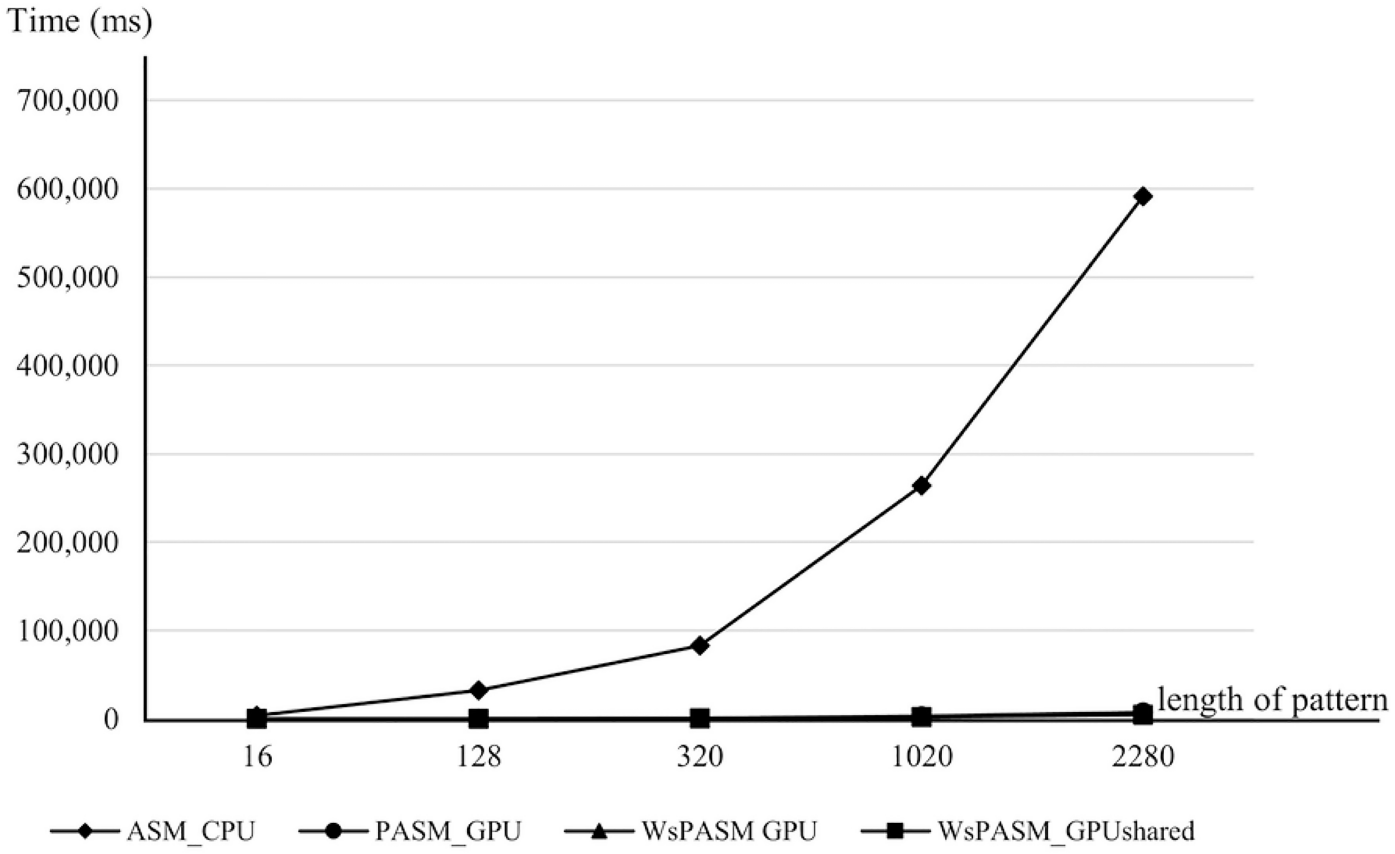
Execution time of ASM_CPU, PASM_GPU, WsPASM_GPU, and WsPASM_GPUshared with various lengths of target patterns.

**Fig 7:**
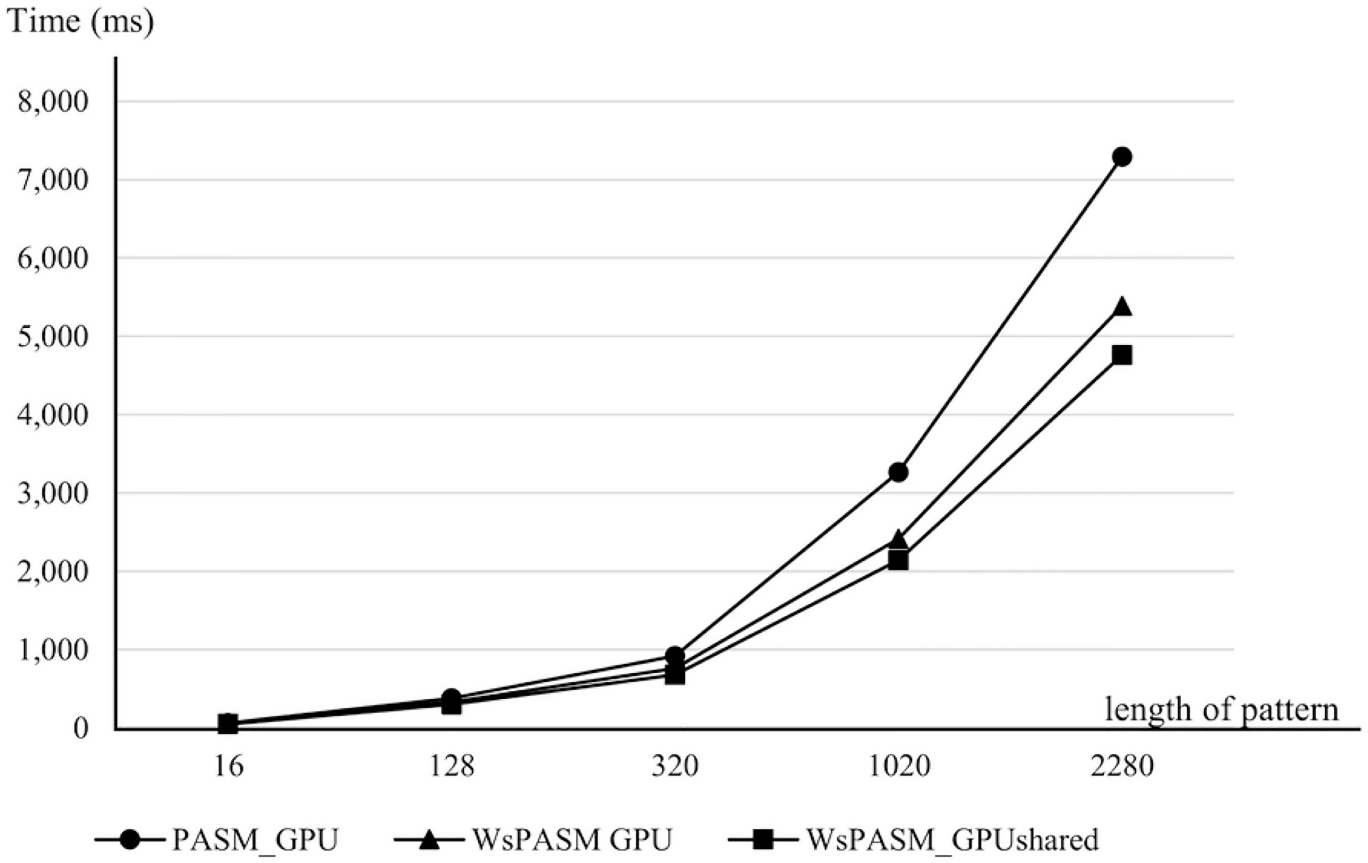
Execution time of PASM_GPU, WsPASM_GPU, and WsPASM_GPUshared with various lengths of target patterns.

Figs 6 and 7 show the execution time of ASM_CPU, PASM_GPU, WsPASM_GPU, and WsPASM_GPUshared with various length of patterns. It is noticed that the execution time was increased as the size of pattern was increased. Therefore, the performance of these algorithms decreased with increasing pattern length. Fig 6 shows the execution time of ASM_CPU with other approaches on GPU. Parallel algorithms on GPUs achieved speedup ranging from 62.38 to 122.64 times, compared to CPU approach. Fig 7 shows the execution time comparison of parallel algorithms on GPUs. WsPASM_GPUshared achieved speedup ranging from 25.89% to 53.11%, compared to previous approach (PASM_GPU).

## Conclusion

This paper proposes a parallel algorithm and its implementation for ASM with *k*-differences on GPUs. The key idea of the proposed algorithm is based on the use of warp-shuffle operations to eliminate the assess of global memory or shared memory. Moreover, this paper also exploits the memory model of GPUs to optimize the performance the proposed algorithm. With realistic DNA data, the performance of proposed algorithm has enhanced up to 1.53 and 122.64 times, compared to that of the previous parallel approach on GPUs and the sequential algorithm on CPU, respectively. Therefore, the proposed algorithm and its implementation can be used to effectively enhance the performance of ASM.
